# Frailty-free life expectancy and its association with socio-economic characteristics: an analysis of the English Longitudinal Study of Ageing cohort study

**DOI:** 10.1186/s12916-025-04112-z

**Published:** 2025-05-09

**Authors:** David R. Sinclair, Asri Maharani, Andrew Kingston, Terence W. O’Neill, Fiona E. Matthews

**Affiliations:** 1 National Institute for Health and Care Research (NIHR) Policy Research Unit in Healthy Ageing, Newcastle University, Newcastle upon Tyne, UK; 2 Division of Nursing, Midwifery and Social Work, School of Health Sciences, Faculty of Biology, Medicine and Health, The University of Manchester, Manchester, UK; 3 National Institute for Health and Care Research (NIHR) Policy Research Unit in Healthy Ageing, The University of Manchester, Manchester, UK; 4 University of Hull, Cottingham Road, Hull, UK

**Keywords:** Frail, Pre-frail, Inequalities, Wealth, Deprivation, Education, Marital status, ELSA

## Abstract

**Background:**

Frailty is more prevalent in socio-economically disadvantaged groups; however, little is known about how this translates to differences in the number of years people live with and without frailty. We investigate differences in frailty-free and frail life expectancies among population groups stratified by wealth, area deprivation, education and marital status.

**Methods:**

The English Longitudinal Study of Ageing cohort study was used to follow the frailty trajectories of 15,003 individuals over 18 years. A multi-state model assessed the risk of transitioning between frailty states and death based on socio-economic characteristics. These risks were translated into state-specific life expectancies.

**Results:**

Wealth had the strongest association with frailty-free and frail life expectancies. Increased wealth, reduced deprivation, higher educational attainment and marriage all correlate with increased frailty-free life expectancies and reduced frail life expectancies. At age 50, the wealthiest population quintile can expect to live 11.1 [10.1–12.1] years (women) and 9.8 [8.8–10.8] years (men) longer frailty-free than the poorest population quintile. The wealthiest quintile live less than half the number of years with frailty than the poorest quintile. There is no difference in frailty-free life expectancy between the poorest men and women; however, the wealthiest women have longer frailty-free life expectancies than the wealthiest men.

**Conclusions:**

Large inequalities in frailty-free and frail life expectancies exist across socio-economic groups, with wealth and area deprivation the most important socio-economic determinants. Narrowing these inequalities may extend frailty-free life expectancies more for women than men, suggesting strategies to reduce disparities should consider both socio-economic factors and gender. Care policies should account for the geographical clustering of socio-economically disadvantaged populations. Reducing socio-economic inequalities could increase frailty-free life expectancies and reduce health and social care costs.

**Supplementary Information:**

The online version contains supplementary material available at 10.1186/s12916-025-04112-z.

## Background

As populations age, health and social care demands rise, driven by the prolonged period of poor health that typically accompanies advanced age. In the UK, a growing number of older individuals are choosing to stay in their homes for longer in advanced age, necessitating enhanced community and unpaid care provision support for those with declining health [1, 2]. Among the older population, individuals with frailty, a condition associated with ageing and marked by the gradual loss of physiological reserves across multiple body systems, present particular challenges because they have a greater need for health and social care and experience higher mortality rates than the non-frail population [3–6].

The average number of years a person can expect to spend in good health can be estimated using different health expectancy indicators [7]. Analysis with one indicator, disability-free life expectancy, has found stark differences between socio-economically advantaged and disadvantaged groups in the UK [8–11] and elsewhere [12–15]. Although frailty is another key measure of health in older age, there is limited research examining frailty-free life expectancy across socio-economic groups. Factors like lower area deprivation, greater wealth, higher educational attainment and being married are linked to a reduced prevalence of frailty in England [16–20], but it is unclear whether the gap in frailty risk widens or narrows with age [21]. Estimating frailty-free life expectancies across socio-economic groups will aid in planning for future health and social care use and tailoring interventions.

Frailty prevalence in England is estimated to range from 3 to 14% of the older population, depending on the measure of frailty and age range evaluated [22–24]. Demographic and socio-economic determinants are also associated with frailty. Older women are typically frailer than older men, and higher deprivation, lower wealth and lower educational attainment are linked to frailty [16, 24–26]. Despite its widespread use as a measure of older people’s health, few studies have investigated frailty-free life expectancy.

A cross-sectional study of 15 European Union countries estimated frailty-free life expectancy at the age of 70 [27]. For men, this ranged from 4.9 to 13.5 years and for women it ranged from 5.2 to 14.4 years. Additionally, frail life expectancy stood at 0.1–1.8 years for men and 0.4–5.5 years for women. A cross-sectional French study found similar frail life expectancies at age 70: 1.0–1.5 years (men) and 3.0–3.8 years (women) [28]. A longitudinal study using data from the USA reported that increased frailty-free life expectancy was associated with increased education and wealth [29], with a similar result to a smaller study over two waves in São Paulo, Brazil. This Brazilian investigation found that people with higher educational attainment have longer frailty-free life expectancies than those with lower attainment [30]. A longitudinal Swedish study found that frail life expectancies are increasing [31].

The increasing desire for ageing in place has highlighted the need for a more comprehensive understanding of frailty and its consequences. Most existing studies are cross-sectional and geographically specific; the role of socio-economic determinants on frailty-free life expectancies remains inadequately explored using longitudinal data.

Our study uses longitudinal data collected over 18 years to investigate frailty-free and frail life expectancies and their associations with demographic and socio-economic status. To our knowledge, this is the first study to consider area deprivation and marital status and the first to use longitudinal data to investigate socio-economic associations with frailty-free and frail life expectancies in a European setting.

This study aims to calculate frailty-free and frail life expectancies and investigate their associations with socio-economic factors.

## Methods

### Study population

We sourced data from the English Longitudinal Study of Ageing (ELSA) [32]. ELSA is a prospective cohort study representative of people aged 50 and over in England and is a sister study to the US-based Health and Retirement Study [33]. ELSA participants are surveyed on demographic, economic and health measures every 2 years. Refreshment samples are periodically added to ELSA to maintain the representativeness of the ELSA cohort. ELSA does not recruit care home residents; however, individuals who relocate to care homes remain eligible for participation in subsequent waves. We used data from ELSA core members, including refreshment samples. Detailed information on ELSA recruitment has been described previously [34].

ELSA participants who do not appear in at least two survey waves or are not known to have died after appearing in one wave were excluded due to a lack of longitudinal data. We used longitudinal data from 15,003 unique participants covering waves 1–9 (2002–2019).

### Frailty

A frailty index was generated using 60 deficits found in ELSA, using the index constructed by Maharani et al. [16] (see Additional file 1: S1 Table for the list of deficits). This index was developed following the standard procedure for creating a frailty index [35]. Individuals were classified as robust, pre-frail or frail at each wave depending on the proportion of deficits they reported: robust ≤ 0.08; 0.08 < pre-frail < 0.25; frail ≥ 0.25, following established cut-points [36]. While the frailty index is a continuous measure, prior research indicates that these categorical thresholds are associated with both institutionalisation and mortality [36, 37].

### Socio-economic factors

Frailty-free, frail and total life expectancies were calculated and conditioned on four socio-demographic and economic factors: wealth, area deprivation, educational attainment and marital status. Wealth was measured by net household financial wealth, split into quintiles. Area deprivation was measured by the English Index of Multiple Deprivation (IMD) quintiles [38]. IMD is the official measure of deprivation in England; it divides the country into small areas of 1000–3000 people each and ranks them based on factors including employment, education, crime, housing and the environment. Unlike wealth, which reflects an individual’s household, IMD describes the broader socio-economic conditions of the area a person lives in.

Educational attainment was stratified into three levels: less than high school level (< 10 years of education), high school level (10–11 years) and more than high school level (≥ 12 years). Marital status was categorised as married and not married, with the latter including people who were formerly married (e.g. widowed or divorced).

Wealth, educational attainment and marital status were self-reported, while area deprivation was derived from participant postcode. The handling of missing data is discussed in Additional file 1: S2 Supplemental methods and S2 Table [39].

### Statistical analysis

**Fig. 1:**
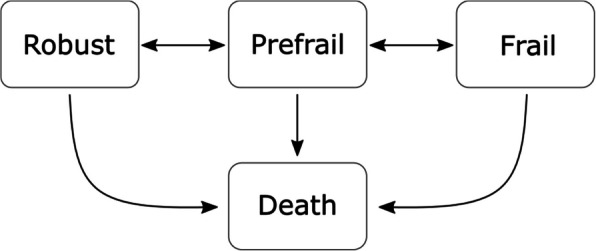
State transition diagram for frailty model. Individuals can transition between adjacent frailty states or to death

We used a continuous time Markov multi-state model to calculate transition intensities related to pre-frailty and frailty. Multi-state models recreate key stages of a discrete process and calculate the risk of transitions between these stages. We used a four-state model, comprising three frailty states—robust, pre-frail and frail—along with death as the absorbing state (Fig. 1). Transitions to death were allowed from any frailty state. The model assumed individuals could not transition directly from robust-to-frail, or vice-versa. Instead, individuals transitioned, at least briefly, through the pre-frail state (this transition was assumed if it was not observed in the panel data). This approach simplified the model by reducing the number of parameters, thereby lowering the computational demands required for its implementation. We also applied the Markov assumption (that the probability of a transition depends only on an individual’s current state) to simplify the model. The multi-state models were run using the *msm* package (*CRAN.R-project.org/package* = *msm*) in *R* (version 4.2.1).

Life expectancies were calculated from the hazard ratios produced by the multi-state models with the *ELECT* (Estimating Life Expectancies using Continuous Time, *CRAN.R-project.org/package* = *elect*) package in *R*. *ELECT* provides a suite of functions for calculating state-specific and marginal life expectancies. With *ELECT*, the parameters of a fitted continuous time multi-state model are used to simulate state-specific life expectancies; a detailed explanation is provided by Van den Hout [40].

Total life expectancy was estimated along with the expected remaining time spent in specific states. A person’s expected remaining time in the robust and pre-frail states was their frailty-free life expectancy, while their remaining time in the frail state was their frail life expectancy. These periods did not have to be continuous. For example, a person may have had frailty following a fall but then recovered to a pre-frail state after a period of rehabilitation. This person may then encounter frailty again towards the end of their life.

Life expectancies were simulated 1000 times in *ELECT* using a bootstraps approach to evaluate uncertainties. Bootstrapping simulates additional samples from the original data set, each of the same size as the original sample, by randomly selecting observations (with replacement). The distribution of the results from each bootstrapped sample informs the confidence interval of the results.

The multi-state model incorporated three frailty states, rather than two, to more accurately represent the complex dynamics of transitions between frailty states. This approach allowed for a clearer distinction between participants at the highest risk of frailty (i.e. those who had pre-frailty) and those with the lowest risk (i.e. those who were robust).

Separate models were created for men and women, due to possible interaction effects between gender and other covariates. Age, wealth, deprivation and marital status were time-variant variables, while gender and educational attainment were modelled as time-invariant. The multi-state model accounted for changes in wealth quintile, deprivation quintile or marital status from one wave to another when calculating the hazard ratios. Model goodness-of-fit was determined by the Bayesian Information Criterion (BIC), which considers both the difference between observed and expected values and the number of covariates used.

We checked the validity of our model results by comparing our total life expectancy estimates to those produced by the UK Government’s Office for National Statistics [41].

### Sensitivity analysis

To prevent identity disclosure, ELSA limits the precision of participants’ date of birth and death data to approximately 1 year, requiring us to generate exact dates of birth and death from the date range constrained by the data (see Additional file 1: S3 Supplemental methods [9]). We repeated our analysis with different sets of generated dates five times to check if the frailty-free and frail life expectancies were sensitive to the generated dates.

## Results

The characteristics of participants in ELSA wave 1 are shown in Table 1. Most participants were women (54.5%) and married (69.7%). The proportion of participants from the least deprived area quintile was higher (23.9%) than those from the most deprived quintile (14.0%). Thirty-seven percent had less than a high school level of education, while 45.5% had more than a high school. Over half were in the robust frailty state (55.9%). The overwhelming majority of the transitions recorded in ELSA were between adjacent frailty states. Only 1% of transitions recorded participants moving from robust-to-frail or frail-to-robust without an intermediate step (Additional file 1: S4 Table).

**Table 1:** Characteristics of ELSA participants, stratified by frailty state, the first time each participant is measured

Characteristic	Overall (%)		Robust (%)		Pre-frail (%)		Frail (%)	
*All*								
	15,003		8393		5099		1511	
*Gender*								
Men	6831	(45.5)	4253	(50.7)	1997	(39.2)	581	(38.5)
Women	8172	(54.5)	4140	(49.3)	3102	(60.8)	930	(61.5)
*Age*								
50–54	4673	(31.1)	3248	(38.7)	1125	(22.1)	300	(19.9)
55–59	2972	(19.8)	1908	(22.7)	820	(16.1)	244	(16.1)
60–64	2098	(14.0)	1206	(14.4)	689	(13.5)	203	(13.4)
65–69	1896	(12.6)	969	(11.5)	729	(14.3)	198	(13.1)
70–74	1565	(10.4)	589	(7.0)	783	(15.4)	193	(12.8)
75–79	960	(6.4)	303	(3.6)	500	(9.8)	157	(10.4)
80–84	609	(4.1)	135	(1.6)	336	(6.6)	138	(9.1)
85 +	230	(1.5)	35	(0.4)	117	(2.3)	78	(5.2)
*Deprivation quintile*								
1st (most deprived)	2104	(14.0)	798	(9.5)	872	(17.1)	434	(28.7)
2nd	2667	(17.8)	1338	(15.9)	991	(19.4)	338	(22.4)
3rd	3099	(20.7)	1727	(20.6)	1087	(21.3)	285	(18.9)
4th	3539	(23.6)	2151	(25.6)	1120	(22.0)	268	(17.7)
5th (least deprived)	3590	(23.9)	2377	(28.3)	1027	(20.1)	186	(12.3)
*Wealth quintile*								
1st (least wealth)	3233	(22.0)	1374	(16.8)	1191	(23.7)	668	(44.8)
2nd	2724	(18.5)	1289	(15.7)	1084	(21.6)	351	(23.5)
3rd	2815	(19.1)	1608	(19.6)	972	(19.4)	235	(15.8)
4th	2933	(19.9)	1819	(22.2)	956	(19.0)	158	(10.6)
5th (most wealth)	3002	(20.4)	2106	(25.7)	816	(16.3)	80	(5.4)
*Marital status*								
Not married	4552	(30.3)	1995	(23.8)	1840	(36.1)	717	(47.5)
Married	10,448	(69.7)	6396	(76.2)	3258	(63.9)	794	(52.5)
*Educational attainment*								
Less than high school (< 10 years)	5557	(37.0)	2380	(28.4)	2241	(43.9)	936	(61.9)
High school (10–11 years)	2613	(17.4)	1604	(19.1)	841	(16.5)	168	(11.1)
College or higher (≥ 12 years)	6833	(45.5)	4409	(52.5)	2017	(39.6)	407	(26.9)

Only includes participants who appear in at least one subsequent wave of ELSA or are known to have died

Our models found that a woman aged 50 years can expect 28.8 frailty-free years [95% confidence interval: 28.4–29.2 years] and 7.1 [6.8–7.4] years with frailty (Table 2). A 50-year-old man can expect a similar number of frailty-free years, 28.3 [27.9–28.7], but only 4.3 [4.1–4.5] years with frailty (Table 3). Men aged 50 will spend a greater proportion of their remaining life frailty-free than women (86.8 [86.0–87.5]% vs 80.2 [79.4–80.9]%). Frailty-free and frail life expectancies for men and women at age 70 are included in Additional file 1: S5 Table.

**Table 2:** Age 50 frailty-free, frail and total life expectancies for women

Characteristic	Frailty-free				Frail				Total	
	**Years**		**Percentage**		**Years**		**Percentage**		**Years**	
*All*										
	28.8	(28.4–29.2)	80.2	(79.4–80.9)	7.1	(6.8–7.4)	19.8	(19.1–20.6)	35.9	(35.5–36.3)
*Wealth quintile*										
1st (least wealth)	23.9	(23.3–24.4)	72.2	(70.7–73.7)	9.2	(8.7–9.8)	27.8	(26.3–29.3)	33.1	(32.4–33.7)
2nd	26.8	(26.3–27.1)	77.5	(76.5–78.2)	7.8	(7.5–8.2)	22.5	(21.8–23.5)	34.6	(34.1–35.0)
3rd	29.6	(29.2–30.0)	82.0	(81.2–82.7)	6.5	(6.2–6.8)	18.0	(17.3–18.8)	36.1	(35.7–36.5)
4th	32.4	(31.8–32.9)	85.9	(84.8–86.8)	5.4	(5.0–5.7)	14.3	(13.2–15.2)	37.7	(37.1–38.3)
5th (most wealth)	35.0	(34.2–35.7)	88.8	(87.9–90.1)	4.3	(3.8–4.8)	10.9	(9.9–12.1)	39.4	(38.4–40.1)
*Deprivation quintile*										
1st (most deprived)	24.5	(23.8–25.1)	73.1	(71.5–74.8)	9.0	(8.4–9.5)	26.9	(25.2–28.5)	33.5	(32.8–34.1)
2nd	26.5	(26.0–26.9)	76.6	(75.5–77.6)	8.1	(7.7–8.5)	23.4	(22.4–24.5)	34.6	(34.1–35.1)
3rd	28.4	(28.0–28.7)	79.6	(78.8–80.3)	7.3	(7.0–7.6)	20.4	(19.7–21.2)	35.7	(35.3–36.1)
4th	30.4	(29.9–30.8)	82.4	(81.5–83.1)	6.5	(6.2–6.9)	17.6	(16.9–18.5)	36.9	(36.4–37.3)
5th (least deprived)	32.3	(31.5–32.9)	85.0	(83.7–85.9)	5.8	(5.3–6.2)	15.3	(14.1–16.3)	38.0	(37.3–38.7)
*Educational quintile*										
Less than high school (< 10 years)	29.0	(28.5–29.3)	80.1	(79.3–80.9)	7.2	(6.9–7.5)	19.9	(19.1–20.7)	36.2	(35.7–36.5)
High school (10-11 years)	31.4	(30.8–32.0)	82.8	(81.8–84.2)	6.4	(5.9–6.9)	16.9	(15.8–18.2)	37.9	(37.1–38.5)
College or higher (≥ 12 years)	33.9	(32.8–34.9)	85.6	(84.1–87.3)	5.7	(5.0–6.4)	14.4	(12.7–15.9)	39.6	(38.3–40.6)
*Marital status*										
Not married	27.3	(26.7–27.8)	77.8	(76.7–78.8)	7.8	(7.4–8.2)	22.2	(21.2–23.3)	35.1	(34.4–35.6)
Married	30.2	(29.6–30.8)	82.5	(81.4–83.8)	6.4	(5.9–6.8)	17.4	(16.2–18.6)	36.6	(35.9–37.2)

Life expectancies are stratified by socio-economic characteristics. Results are from a univariate analysis of each characteristic

95% confidence intervals in parentheses

**Table 3:** Age 50 frailty-free, frail and total life expectancies for men

Characteristic	Frailty-free				Frail				Total	
	**Years**		**Percentage**		**Years**		**Percentage**		**Years**	
*All*										
	28.3	(27.9–28.7)	86.8	(86.0–87.5)	4.3	(4.1–4.5)	13.2	(12.5–14.0)	32.6	(32.2–33.0)
*Wealth quintile*										
1st (least wealth)	23.4	(22.8–23.9)	79.6	(78.1–81.1)	6.0	(5.6–6.5)	20.4	(18.9–21.9)	29.4	(28.7–30.0)
2nd	25.9	(25.4–26.3)	83.8	(82.9–84.7)	5.0	(4.7–5.3)	16.2	(15.3–17.1)	30.9	(30.4–31.3)
3rd	28.4	(28.0–28.8)	87.4	(86.7–88.1)	4.1	(3.9–4.3)	12.6	(11.9–13.3)	32.5	(32.1–32.9)
4th	30.8	(30.3–31.3)	90.3	(89.6–91.1)	3.3	(3.0–3.6)	9.7	(8.9–10.4)	34.1	(33.6–34.6)
5th (most wealth)	33.2	(32.4–33.8)	92.7	(91.8–93.5)	2.6	(2.3–2.9)	7.3	(6.5–8.2)	35.8	(35.0–36.4)
*Deprivation quintile*										
1st (most deprived)	23.9	(23.1–24.5)	80.7	(79.3–82.3)	5.7	(5.2–6.2)	19.3	(17.7–20.7)	29.6	(28.8–30.2)
2nd	25.9	(25.4–26.3)	83.8	(82.8–84.6)	5.0	(4.7–5.3)	16.2	(15.4–17.2)	30.9	(30.4–31.3)
3rd	27.9	(27.4–28.2)	86.4	(85.5–87.0)	4.4	(4.2–4.7)	13.6	(13.0–14.5)	32.3	(31.8–32.6)
4th	29.8	(29.3–30.3)	88.4	(87.8–89.2)	3.9	(3.6–4.1)	11.6	(10.8–12.2	33.7	(33.2–34.1)
5th (least deprived)	31.8	(31.1–32.4)	90.6	(89.5–91.4)	3.3	(3.0–3.7)	9.4	(8.6–10.5)	35.1	(34.4–35.7)
*Educational quintile*										
Less than high school (< 10 years)	28.2	(27.7–28.6)	86.5	(85.6–87.1)	4.4	(4.2–4.7)	13.5	(12.9–14.4)	32.6	(32.1–33.0)
High school (10-11 years)	30.2	(29.5–30.8)	88.6	(87.6–89.6)	3.9	(3.5–4.2)	11.4	(10.4–12.4)	34.1	(33.3–34.7)
College or higher (≥ 12 years)	32.2	(31.1–33.1)	90.7	(89.4–91.9)	3.3	(2.8–3.8)	9.3	(8.1–10.6)	35.5	(34.3–36.5)
*Marital status*										
Not married	25.6	(24.8–26.2)	84.8	(83.5–86.0)	4.6	(4.2–5.1)	15.2	(14.0–16.5)	30.2	(29.4–30.8)
Married	29.3	(28.7–29.7)	87.2	(86.5–88.1)	4.3	(4.0–4.6)	12.8	(11.9–13.5)	33.6	(33.0–34.0)

Life expectancies are stratified by socio-economic characteristics. Results are from a univariate analysis of each characteristic

95% confidence intervals in parentheses

Wealth is the most strongly associated with frailty-free life expectancy and provides a better model fit than area deprivation, marital status and educational attainment according to model BIC (Additional file 1: S6 Table a–k). When combining multiple covariates, a person’s wealth and area deprivation have a stronger association with frailty-free life expectancy than wealth alone based on their BIC.

### Wealth

**Fig. 2:**
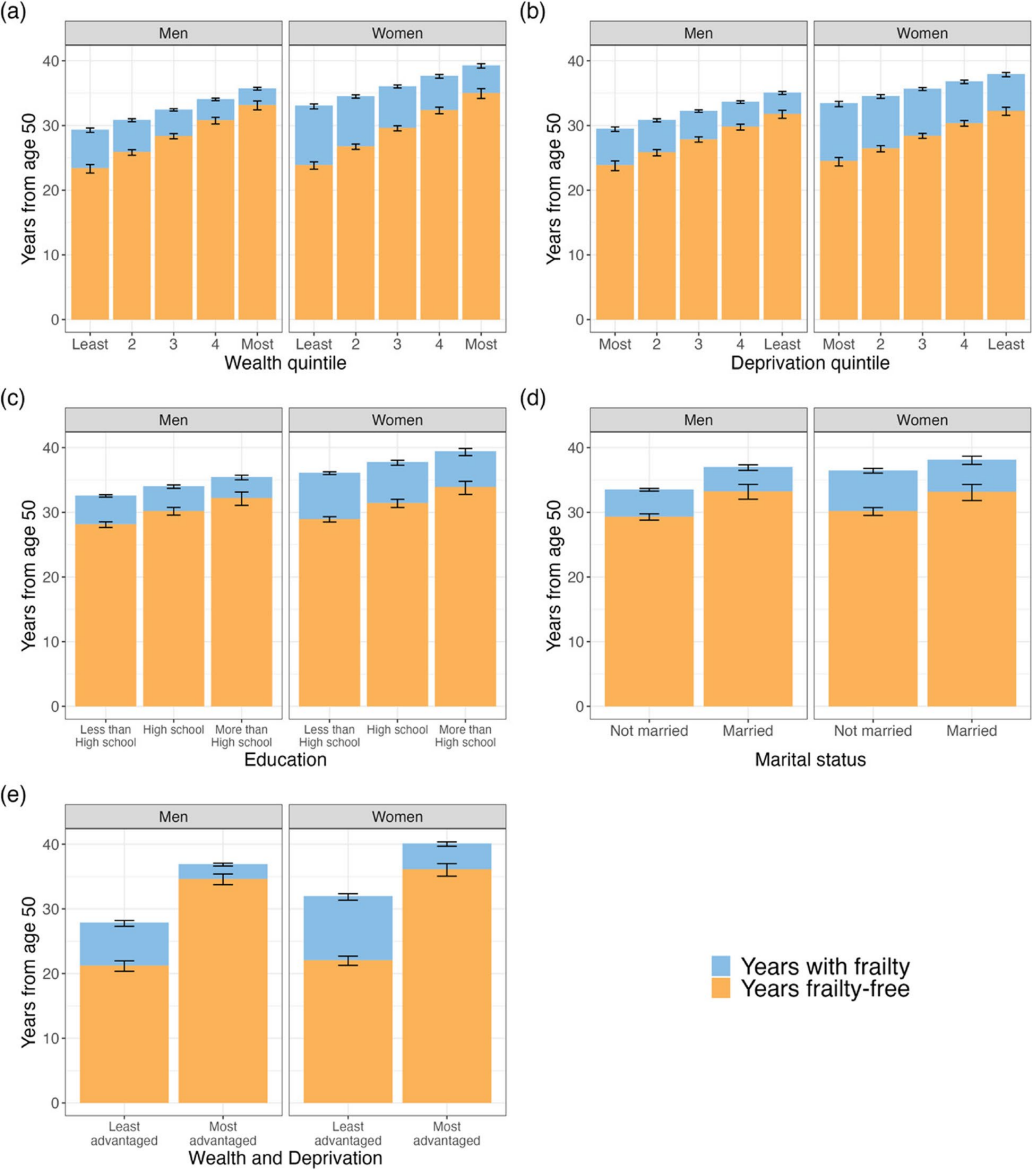
Frailty-free and frail life expectancies stratified by socio-economic groups. Frailty-free and frail life expectancies of 50-year-old men and women, stratified by **a** wealth; **b** area deprivation; **c** educational attainment; **d** marital status; and **e** wealth and area deprivation quintiles. Wealth is measured by the net household financial wealth quintile. Area deprivation is measured by the English Index of Multiple Deprivation quintile. Education is stratified into less than high school (< 10 years of education), high school (10–11 years) and more than high school (≥ 12 years). Marital status is stratified into married and not married. **e** contrasts men and women who are in the wealthiest quintile and live in the least deprived area quintile, against men and women who are in the least wealthy quintile and live in the most deprived areas

Greater wealth is associated with increased frailty-free and total life expectancy, as well as shorter frail life expectancy for both men and women (Fig. 2a and Tables 2–3). A 50-year-old woman in the wealthiest quintile can expect 11.1 [10.1–12.1] years more frailty-free than her counterpart in the least wealthy quintile, with a 9.8 [8.8–10.8] year difference between the most and least wealthy men. In relative terms, men aged 50 spend a greater percentage of their remaining life frailty-free than equivalently wealthy women across all wealth quintiles.

In the lowest wealth quintile, women and men had a similar frailty-free life expectancy at age 50 (women: 23.9 [23.3–24.4] years, men: 23.4 [22.8–23.9] years). However, women in higher wealth quintiles have longer frailty-free life expectancies than men, with the gap increasing with wealth (women at age 50 in the wealthiest quintile: 35.0 [34.2–35.7] years; men: 33.2 [32.4–33.8] years).

Frail life expectancy for the least wealthy was more than double that of the wealthiest (women: 9.2 [8.7–9.8] vs 4.3 [3.8–4.8] years; men: 6.0 [5.6–6.5] vs 2.6 [2.3–2.9] years). While the wealthiest women and men have shorter frail life expectancies, their total life expectancies are notably greater than for the least wealthy women and men (6.3 [5.1–7.5] years longer for women, 6.4 [5.3–7.5] years for men).

Men and women in the least wealthy quintile spend a much greater percentage of their remaining lives with frailty than their wealthiest peers (women: 27.8 [26.3–29.3]% vs 10.9 [9.9–12.1]%, men: 20.4 [18.9–21.9]% vs 7.3 [6.5–8.2]%).

### Deprivation

Lower deprivation followed a similar pattern to increased wealth: greater frailty-free life expectancy and shorter frail life expectancy (Fig. 2b and Table 2). The associations were less strong than wealth, with a smaller difference between the most and least deprived than the most and least wealthy (e.g. 7.8 [6.7–8.9] additional years of frailty-free life expectancy between the most and least deprived 50-year-old women, compared to 11.1 [10.1–12.1] additional years between the most and least wealthy women).

We found no evidence for differences in frailty-free life expectancies for men and women aged 50 in the same deprivation quintile (at the 95% confidence limit); however, in relative terms men consistently spend a greater percentage of their remaining lives frailty-free than women. Women consistently had longer frail life expectancies than men and spent a greater percentage of their lives with frailty.

Total life expectancies were longer for people living in the least deprived areas compared to the most deprived areas (women: 4.5 [3.5–5.5] more years, men: 5.5 [4.4–6.6] more years).

### Educational attainment

Higher education attainment also followed the same pattern as wealth and deprivation, albeit with smaller differences (Fig. 2c and Table 2). Fifty-year-old women with college or higher education had 4.9 [3.7–6.1] years greater frailty-free life expectancy than those with less than high school education (33.9 [32.8–34.9] years vs 29.0 [28.5–29.3] years). Fifty-year-old men had a difference of 4.0 [2.8–5.2] years (32.2 [31.1–33.1] years vs 28.2 [27.7–28.6] years).

Total life expectancies were also longer for people with the greatest educational attainment versus those with the least, albeit with a smaller difference than with wealth or deprivation (women: 3.4 [2.0–4.8] more years, men: 2.9 [1.6–4.2] more years).

### Marital status

Married men and women had longer frailty-free life expectancies than unmarried men and women (Fig. 2d and Table 2). Married women also had a shorter frail life expectancy at age 50 than unmarried women (6.4 [5.9–6.8] vs 7.8 [7.4–8.2] years).

Married individuals have larger total life expectancies than unmarried individuals, but the advantage was more pronounced for men. Married men lived an average of 3.4 [2.4–4.4] years longer than unmarried men, compared to a smaller difference of 1.5 [0.5–2.5] years for married women over unmarried women.

There was no difference in frail life expectancy for married and unmarried men (4.6 [4.2–5.1] years vs 4.3 [4.0–4.6] years). However, as married men have larger total life expectancies, unmarried men spend a greater percentage of their lives with frailty.

### Wealth and deprivation

The combination of wealth and area deprivation provided the strongest association with frailty-free life expectancy, based on their BIC scores (Additional file 1: S6 Tables a–k). Frailty-free and frail life expectancies of men and women according to wealth and deprivation quintile at age 50 are shown in Fig. 2e and Table 4. Fifty-year-old women in the most advantaged wealth and deprivation quintiles had 14.0 [12.7–15.3] years longer frailty-free than women in the least advantaged quintiles, with 36.1 [35.1–37.0] years versus 22.1 [21.3–22.7] years, respectively. A similarly large difference was observed for men, with those in the most advantaged quintiles having 13.3 [12.0–14.6] years longer frailty-free life expectancy than men in the least advantaged quintiles—34.6 [33.7–35.4] years versus 21.3 [20.4–22.0] years.

**Table 4:** Age 50 frailty-free, frail and total life expectancies in years for women and men

	Women (CI)				Men (CI)			
	Years		Percentage		Years		Percentage	
*Most advantaged*								
Frailty-free LE	36.1	(35.1–37.0)	90.0	(89.6–90.7)	34.6	(33.7–35.4)	93.8	(93.4–94.3)
Frail LE	4.0	(3.6–4.5)	10.0	(9.3–10.4)	2.3	(2.0–2.6)	6.2	(5.7–6.6)
Total LE	40.1	(39.0–41.0)	–	–	36.9	(36.0–37.7)	–	–
*Least advantaged*								
Frailty-free LE	22.1	(21.3–22.7)	69.3	(67.4–70.2)	21.3	(20.4–22.0)	76.6	(74.5–77.5)
Frail LE	9.9	(9.3–10.6)	31.0	(29.8–32.6)	6.6	(6.1–7.3)	23.7	(22.5–25.5)
Total LE	31.9	(31.2–32.7)	–	–	27.8	(27.0–28.7)	–	–

The most advantaged group are people in both the most wealthy and least deprived quintiles. The least advantaged group are people in both the least wealthy and most deprived quintiles

*LE*, Life expectancy, *CI*, 95% Confidence interval

At age 50, the women in both the wealthiest and least deprived quintiles have 5.9 [5.2–6.6] years shorter frail life expectancies than the women in the poorest and most deprived quintiles, with a gap of 4.3 [3.7–4.9] years for men. Total life expectancy for the wealthiest and least deprived women is 8.2 [6.9–9.5] years longer than the poorest and most deprived women, compared to 9.1 [7.9–10.3] years for men. As a proportion of remaining life, the least advantaged men and women spend three-to-four times the amount of time with frailty as the most advantaged men and women.

### Hazard ratios

As age increased, frailty incidence rose and recovery declined across all models (Additional file 1: S6 Tables a–k). Higher wealth, lower area deprivation, greater educational attainment and marriage were all associated with a lower risk of greater frailty and a higher chance of recovering to a lower frailty state. In the best-fitting model, which uses wealth and area deprivation as covariates (Additional file 1: S6 Table g), wealth’s association with frailty transitions was similar for men and women. However, it differed for transitions to death. For women, each increase in wealth quintile reduced the risk of death only from the pre-frail state (hazard ratio, HR: 0.85 [0.76–0.95]), but for men it reduced the risk only from the robust state (HR: 0.81 [0.68–0.95]). Less deprived areas are associated with a reduced risk of death only for robust men (HR: 0.78 [0.66–0.93]).

### Sensitivity analysis

Repeating the analysis five times with different generated precise birth and death dates did not have a significant impact on the model results. The estimated total life expectancies for men and women aged 50–100 were similar to those from the UK Office for National Statistics (Additional file 1: S7 Figure [42]), with a standard error of the estimate of 0.54 years for men and 0.82 years for women.

## Discussion

Using longitudinal data, our study found that greater wealth, lower deprivation, more education and being married are all associated with longer frailty-free life expectancies and shorter frail life expectancies. Socio-economically advantaged groups spend a greater proportion of their lives without frailty compared to their socio-economically disadvantaged peers. The gap between the wealthiest and poorest is such that, at age 50, the wealthiest men and women have longer frailty-free life expectancies than the total life expectancy of the poorest.

Across socio-economic groups, women typically have longer frailty-free life expectancies than men, though men spend a greater proportion of their lives frailty-free. However, among the least wealthy, the most deprived, or those with the lowest educational attainment, frailty-free life expectancy is similar between men and women. Despite this, disadvantaged women aged 50 will typically spend 7% more of their remaining years living with frailty compared to equally disadvantaged men, due to their longer total life expectancies.

The potential mechanisms linking greater wealth, lower area deprivation and more years of education to longer frailty-free life expectancies include improved access to health care, healthier behaviours, favourable psychosocial exposures and the relationship between education and cognitive functioning [43]. Marriage has been found to promote positive health behaviours [44] and healthier individuals are more likely to marry and remain married [45]. These mechanisms, rooted in the socio-economic and cultural context of England, highlight the multifaceted ways in which socio-economic factors influence health trajectories and life expectancy outcomes.

Our results are consistent with prior US and Brazilian studies which found similar benefits associated with wealth and education [29, 30]. Like the US study, we found wealth to be the strongest influence on time spent frailty-free and with frailty, suggesting the importance of wealth in frailty-free life expectancy transcends national borders.

The US study found that women and men aged 70 in their least advantaged group (lowest wealth tertile and less than a high school education) can expect 3.8 [4.5–4.1] and 4.3 [3.8–4.7] frailty-free years, respectively [29]. In São Paulo, Brazil, the least advantaged 70-year-olds (0 years of education) can expect 11.7 [10.6–12.8] and 9.1 [7.9–10.3] frailty-free years, respectively [30]. England falls between these two, with the least advantaged 70-year-old women and men estimated to have 8.0 [7.6–8.4] and 7.9 [7.4–8.3] frailty-free years, respectively (Additional file 1: S5 Table c).

The estimated frailty-free life expectancies are also consistent with those from two cross-sectional EU studies (this comparison is at age 70, the age used in the EU studies) [27, 28]. However, our frail life expectancies are longer than the EU study found. While the geographical differences may partly reflect variations in frailty measures across studies, they also suggest that broader health determinants, beyond those studied examined here, influence frailty-free life expectancies.

The total life expectancies estimated by our model are similar to those from the Office for National Statistics life tables [41], suggesting our model is robust. A small difference emerges at the oldest ages; this may be due to cohorts in longitudinal studies tending to be healthier than the general population [9].

Our findings suggest that gains in total life expectancy do not inevitably lead to more years living with frailty. This implies that extending frailty-free life expectancy, by delaying the onset of frailty, slowing its progression or improving recovery from frailty, may compress the period of frailty into a shorter duration at the end of life, thereby potentially reducing demand for health and social care.

Individuals with frailty have higher health and social care utilisation and costs [46, 47]. Frailty-free and frail life expectancies are strongly associated with socio-economic disadvantage. As socio-economic disadvantage is geographically clustered in England, especially along a north–south divide, our results suggest that health and social care planning should account for uneven demands on these services arising from longer frail life expectancies in socio-economically disadvantaged areas. In particular, deprived areas with low wealth should be prioritised, as these areas will have populations living with frailty for longer.

The similar frailty-free life expectancy between the poorest men and women highlights the impact of socio-economic status on health outcomes. Socio-economic disadvantage is associated with numerous risk factors, including poor nutrition and poor health behaviours [48–51]. These factors may overwhelm gender-specific health differences, resulting in similar frailty-free life expectancies for poor men and women. Conversely, richer men do not experience the same extent of health advantage as richer women, with differences in health behaviour or diet potentially contributing to this gender gap in frailty-free life expectancy.

This suggests that strategies to reduce disparities in frailty-free life expectancy should consider both socio-economic factors and gender. Among poorer populations, interventions should seek to mitigate the adverse health impacts of poverty, whereas among wealthier populations, interventions should focus on gender-specific differences. However, interventions which prioritise reducing frail life expectancies, rather than increasing frailty-free life expectancies, should consider that gender differences in frail life expectancies are more pronounced in the most deprived areas.

### Strengths and limitations

The strengths of this work include the application of a large, longitudinal dataset collected over 18 years with a nationally representative cohort to estimate frail and frailty-free life expectancies. Our analysis stratifies frail and frailty-free life expectancies by four socio-economic characteristics.

Although ELSA is representative of the socio-demographic characteristics of the English population [52], only a small proportion of participants are non-white. As the study population is reflective of the English population, our results are valid; however, we were unable to investigate any differences in frailty-free and frail life expectancies between ethnic groups. In addition, ELSA does not recruit institution-dwelling participants, although it retains participants who move into care homes. This may bias our results towards healthier individuals. Most refreshment cohorts recruited participants aged 50–55, an age group where few adults live in care homes, limiting this bias [53].

A further limitation is the potential for selection biases in loss-to-follow-up from participants dropping out of the ELSA survey. Loss due to mortality is factored into the model; however there is informative attrition due to participants dropping out for other reasons. Additionally, ELSA participants could schedule their interview for a convenient time, which may reduce the likelihood of capturing health data during short-term acute illnesses.

Our multi-state model used the Markov assumption: the risk of each transition is only dependent on the current state and characteristics, and not any previous data. This standard assumption simplifies the model but does lose some information which could inform risk. Additionally, as ELSA only collects data biennially, we do not have detailed information about what transitions may occur between these time points.

## Conclusions

Greater wealth, living in less deprived areas, higher education attainment and marriage are linked to longer frailty-free life expectancies and shorter frail life expectancies. Wealth has the strongest association. While frailty-free life expectancy is similar for men and women in the lowest socio-economic groups, women in higher socio-economic groups live longer frailty-free than men.

We found large disparities: wealthy 50-year-olds who live in the least deprived areas live 13–14 years longer without frailty than the least wealthy who live in the most deprived areas. Additionally, individuals with low wealth who live in highly deprived areas spend 4–6 more years with frailty. These findings underscore the importance of addressing socio-economic inequalities at both the individual and area level, to promote healthier ageing and inform health and social care policies.

## Supplementary Information

Additional file 1: S1 Table, S2–S3 Supplemental methods, S4–S6 Tables, S7 Figure. S1 Table—Frailty index deficits. S2 Supplemental methods—Missing data. S3 Supplemental methods—Mortality data in ELSA. S4 Table—State transition counts. S5 Table—Hazard ratios and Bayesian Information Criterion (BIC) of models. S6 Table—Frailty-free, frail and total life expectancies at age 70. S7 Figure—Comparison of total life expectancy estimates to Office for National Statistics estimates.

## Data Availability

The datasets analysed during the current study are available from the UK Data Service, (https://beta.ukdataservice.ac.uk/datacatalogue/series/series?id=200011). Restrictions apply to the availability of the mortality data for deaths after ELSA wave 6, which were used under license for the current study, and so are not publicly available.

## Abbreviations

ELSA: English Longitudinal Study of Ageing
IMD: English Index of Multiple Deprivation
ELECT: Estimating Life Expectancies using Continuous Time
BIC: Bayesian Information Criterion
HR: Hazard ratio
