# Supplementary material for: Frailty-free life expectancy and its association with socio-economic characteristics: an analysis of the English Longitudinal Study of Ageing cohort study
Source: BMC Med. 2025 May 9;23:276. doi: 10.1186/s12916-025-04112-z (PMC12065162; doi:10.1186/s12916-025-04112-z)
Supplement: Supplementary file 1 — Additional file 1: S1 Table, S2–S3 Supplemental methods, S4–S6 Tables, S7 Figure. S1 Table—Frailty index deficits. S2 Supplemental methods—Missing data. S3 Supplemental methods—Mortality data in ELSA. S4 Table—State transition counts. S5 Table—Hazard ratios and Bayesian Information Criterion (BIC) of models. S6 Table—Frailty-free, frail and total life expectancies at age 70. S7 Figure—Comparison of total life expectancy estimates to Office for National Statistics estimates. [file 12916_2025_4112_MOESM1_ESM.docx]

**Additional file 1**

Frailty-free life expectancy and its association with socio-economic characteristics: an analysis of the English Longitudinal Study of Ageing cohort study

| **S1 Table**: Frailty index deficits | p2 |
| --- | --- |
| **S2 Supplemental methods**: Missing data | p4 |
| **S3 Supplemental methods**: Mortality data in ELSA | p5 |
| **S4 Table**: State transition counts | p6 |
| **S5 Tables a-c**: Frailty-free, frail and total life expectancies at age 70 | p7 |
| **S6 Tables a-k**: Hazard ratios and Bayesian Information Criterion (BIC) of models | p10 |
| **S7 Figure**: Comparison of total life expectancy estimates to Office for National Statistics estimates | p17 |

S1 Table A: Frailty index deficits

Participant frailty score is calculated by summing their assigned values and dividing by the number of deficits (n=60).

|  | | | | | | | |
| --- | --- | --- | --- | --- | --- | --- | --- |
|  | **Description** | **Assigned value** | | | | | |
| 1 | Difficulty with walking 100 yards | No=0 | Yes=1 |  |  |  |  |
| 2 | Difficulty sitting for about two hours | No=0 | Yes=1 |  |  |  |  |
| 3 | Difficulty getting up from a chair after sitting for long periods | No=0 | Yes=1 |  |  |  |  |
| 4 | Difficulty climbing several flights of stairs without resting | No=0 | Yes=1 |  |  |  |  |
| 5 | Difficulty climbing one flight of stairs without resting | No=0 | Yes=1 |  |  |  |  |
| 6 | Difficulty stooping, kneeling, or crouching | No=0 | Yes=1 |  |  |  |  |
| 7 | Difficulty reaching or extending arms above shoulder level | No=0 | Yes=1 |  |  |  |  |
| 8 | Difficulty pulling or pushing large objects like a living room chair | No=0 | Yes=1 |  |  |  |  |
| 9 | Difficulty lifting or carrying weights over 10 pounds, like a heavy bag | No=0 | Yes=1 |  |  |  |  |
| 10 | Difficulty picking up a 5p coin from a table | No=0 | Yes=1 |  |  |  |  |
| 11 | Difficulty dressing, including putting on shoes and socks | No=0 | Yes=1 |  |  |  |  |
| 12 | Difficulty walking across a room | No=0 | Yes=1 |  |  |  |  |
| 13 | Difficulty bathing or showering | No=0 | Yes=1 |  |  |  |  |
| 14 | Difficulty eating, such as cutting up your food | No=0 | Yes=1 |  |  |  |  |
| 15 | Difficulty getting in or out of bed | No=0 | Yes=1 |  |  |  |  |
| 16 | Difficulty using the toilet, including getting up or down | No=0 | Yes=1 |  |  |  |  |
| 17 | Difficulty using a map to figure out how to get around in a strange place | No=0 | Yes=1 |  |  |  |  |
| 18 | Difficulty preparing a hot meal | No=0 | Yes=1 |  |  |  |  |
| 19 | Difficulty shopping for groceries | No=0 | Yes=1 |  |  |  |  |
| 20 | Difficulty making telephone calls | No=0 | Yes=1 |  |  |  |  |
| 21 | Difficulty taking medications | No=0 | Yes=1 |  |  |  |  |
| 22 | Difficulty managing money, (e.g. paying bills and keeping track of expenses) | No=0 | Yes=1 |  |  |  |  |
| 23 | Difficulty doing work around the house or garden | No=0 | Yes=1 |  |  |  |  |
| 24 | Self-reported general health | Excellent=0 | V.good-0.25 | Good=0.5 | Fair=0.75 | Poor=1 |  |
| 25 | Whether respondent has felt depressed much of the time during the past week | No=0 | Yes=1 |  |  |  |  |
| 26 | Whether respondent felt everything they did during the past week was an effort | No=0 | Yes=1 |  |  |  |  |
| 27 | Whether respondent felt their sleep was restless much of the time during the past week | No=0 | Yes=1 |  |  |  |  |
| 28 | Whether respondent was happy much of the time during the past week | Yes=0 | No=1 |  |  |  |  |
| 29 | Whether respondent felt lonely much of the time during the past week | No=0 | Yes=1 |  |  |  |  |
| 30 | Whether the respondent enjoyed life much of the time during the past week | Yes=0 | No=1 |  |  |  |  |
| 31 | Whether respondent felt sad much of the time during the past week | No=0 | Yes=1 |  |  |  |  |
| 32 | Whether respondent could not get going much of the time during the past week | No=0 | Yes=1 |  |  |  |  |
| 33 | High blood pressure or hypertension (self-reported) | No=0 | Yes=1 |  |  |  |  |
| 34 | Angina (self-reported) | No=0 | Yes=1 |  |  |  |  |
| 35 | Heart attack (including MI or coronary thrombosis) (self-reported) | No=0 | Yes=1 |  |  |  |  |
| 36 | Congestive heart failure (self-reported)  An abnormal heart rhythm (self-reported) | No=0 | Yes=1 |  |  |  |  |
| 37 | An abnormal heart rhythm (self-reported) | No=0 | Yes=1 |  |  |  |  |
| 38 | Diabetes or high blood sugar (self-reported) | No=0 | Yes=1 |  |  |  |  |
| 39 | A stroke (cerebral vascular disease) (self-reported) | No=0 | Yes=1 |  |  |  |  |
| 40 | Chronic lung disease such as chronic bronchitis or emphysema (self-reported) | No=0 | Yes=1 |  |  |  |  |
| 41 | Asthma (self-reported) | No=0 | Yes=1 |  |  |  |  |
| 42 | Arthritis (including osteoarthritis, or rheumatism) (self-reported) | No=0 | Yes=1 |  |  |  |  |
| 43 | Osteoporosis, sometimes called thin or brittle bones (self-reported) | No=0 | Yes=1 |  |  |  |  |
| 44 | Cancer or a malignant tumour (excluding minor skin cancers) (self-reported) | No=0 | Yes=1 |  |  |  |  |
| 45 | Parkinson's disease (self-reported) | No=0 | Yes=1 |  |  |  |  |
| 46 | Any emotional, nervous or psychiatric problems (self-reported) | No=0 | Yes=1 |  |  |  |  |
| 47 | Alzheimer's disease (self-reported) | No=0 | Yes=1 |  |  |  |  |
| 48 | Dementia, organic brain syndrome, senility or any other serious memory impairment (self-reported) | No=0 | Yes=1 |  |  |  |  |
| 49 | Self-reported eyesight function (while using lenses, if appropriate) | Excellent=0 | V.good=0.2 | Good=0.4 | Fair=0.6 | Poor=0.8 | Blind=1 |
| 50 | Self-reported hearing function (while using hearing aid if appropriate) | Excellent=0 | V.good=0.25 | Good=0.5 | Fair=0.75 | Poor=1 |  |
| 51 | Whether respondent has fallen down at all / in last year / in last 2 years | No=0 | Yes=1 |  |  |  |  |
| 52 | Whether respondent has fractured hip ever / in last 2 years | No=0 | Yes=1 |  |  |  |  |
| 53 | Whether respondent has had joint replacement ever | No=0 | Yes=1 |  |  |  |  |
| 54 | Whether respondent has had any pain whilst walking | No=0 | Yes=1 |  |  |  |  |
| 55 | Identify today's date: day of month | No=0 | Yes=1 |  |  |  |  |
| 56 | Identify today's date: month | No=0 | Yes=1 |  |  |  |  |
| 57 | Identify today's date: year | No=0 | Yes=1 |  |  |  |  |
| 58 | Identify the day of the week | No=0 | Yes=1 |  |  |  |  |
| 59 | Immediate word recall (sample organized into quartiles) | 1^st^ quartile=0 | 2nd=0.3 | 3rd=0.6 | 4^th^ quartile=1 |  |  |
| 60 | Delayed word recall (sample organized into quintiles) | 1^st^ quintile=0 | 2nd=0.25 | 3rd=0.5 | 4th=0.75 | 5^th^ quintile=1 |  |

S2 Supplemental methods: Missing data

A multi-state model does not require participants to report data in every wave. When participants are lost to follow up or miss a wave, the model finds the best fit for the risk of transitioning between states based on the data that is present.

When a socio-economic factor was not reported for a participant in a given wave, the value reported from the nearest prior or subsequent wave was used, with priority given to prior waves. If no value was reported at any wave for a participant, that participant was excluded from models which included that socio-economic factor only. Linkage to Index of Multiple Deprivation quintile is only available for waves 1-8 only available at the time of analysis [38]. IMD quintiles in wave 9 were assumed to match those from wave 8 (or an earlier wave in the rare cases where it was not available in wave 8).

| **S2 Table:** Missing data by wave and covariate | | | | | | | | | | |
| --- | --- | --- | --- | --- | --- | --- | --- | --- | --- | --- |
|  | **Wave** | | | | | | | | | |
| **Covariate** | **1 (%)** | | **2 (%)** | | **3 (%)** | | **4 (%)** | | **5 (%)** | |
| **Wealth** | 199 | (1.7) | 122 | (1.3) | 242 | (2.6) | 292 | (2.7) | 227 | (2.3) |
| **Deprivation** | 0 | (0.0) | 8 | (0.1) | 9 | (0.1) | 23 | (0.2) | 36 | (0.4) |
| **Education** | 0 | (0.0) | 0 | (0.0) | 0 | (0.0) | 0 | (0.0) | 0 | (0.0) |
| **Married** | 2 | (0.0) | 1 | (0.0) | 1 | (0.0) | 1 | (0.0) | 2 | (0.0) |
|  |  | | | | | | | | | |
|  | **6 (%)** | | **7 (%)** | | **8 (%)** | | **9 (%)** | |  |  |
| **Wealth** | 236 | (2.3) | 246 | (2.6) | 145 | (1.7) | 155 | (1.8) |  |  |
| **Deprivation** | 34 | (0.3) | 39 | (0.4) | 44 | (0.5) | 8543 | (100) |  |  |
| **Education** | 0 | (0.0) | 0 | (0.0) | 0 | (0.0) | 0 | (0.0) |  |  |
| **Married** | 2 | (0.0) | 2 | (0.0) | 4 | (0.0) | 0 | (0.0) |  |  |

Deprivation quintiles were not available for wave 9 and were imputed by last observation carried forward.

S3 Supplemental methods: Mortality data in ELSA

Mortality dates were sourced from three datasets connected with ELSA, as the standard dataset does not include dates of death for all participants. For participants who died after wave 6, the year of death for ELSA participants was obtained by linked civil registration data (accessed with permission via the ELSA team). For participants who died before wave 6, 21% are associated with end-of-life interviews which provide participant year of death. The remaining 79% who died before wave 6 do not have a year of death, but a wave of death. The wave of death provides an upper and lower limit for the date of death. These limits vary by the wave of death, but each covers a 1-2 year period. Once upper and lower limits for the year of death were determined, a date of death was randomly generated by picking a day within the boundaries for each participant (using a uniform distribution). Exact dates of birth were also drawn from a uniform distribution of days within each participant’s birth year.

To ensure the robustness of our findings, we generated five additional sets of birth and death dates and re-ran our models to check its sensitivity to date of birth and death. The model outputs did not change significantly across the generated data sets.

We also validated our results by comparing our estimated total life expectancies to those from the Office for National Statistics (Additional file 1: Figure S7). We found a standard error of the estimate between our model’s total life expectancies and the ONS’s estimates to be 0.54 years for men and 0.82 years for women. This difference may be due to cohorts in longitudinal studies tending to be healthier than the general population [41].

**S4 Table**: **State transition counts**

Includes when participants remain in the same state from one measurement to the next.

| **From** | **To** | | | |
| --- | --- | --- | --- | --- |
|  | **Robust** | **Prefrail** | **Frail** | **Death** |
| **Robust** | 12478 | 3745 | 97 | 106 |
| **Prefrail** | 2563 | 10976 | 1957 | 437 |
| **Frail** | 18 | 1063 | 3827 | 596 |

Only 0.3% of state data and 1% of transitions involved direct movement between the robust and frail states.

S5 Table: Frailty-free, frail and total life expectancies at age 70

| **S5 Table a: Age 70 frailty-free, frail and total life expectancies for women.** | | | | | | | | | | |
| --- | --- | --- | --- | --- | --- | --- | --- | --- | --- | --- |
|  | **Frailty-Free** | | | | **Frail** | | | | **Total** | |
| **Characteristic** | **Years** | | **Percentage** | | **Years** | | **Percentage** | | **Years** | |
| *All* | | | | | | | | | | |
|  | 11.8 | (11.5-12.1) | 66.3 | (64.7-67.4) | 6.1 | (5.8-6.3) | 34.3 | (32.6-35.3) | 17.8 | (17.5-18.2) |
| *Wealth quintile* | | | | | | | | | | |
| 1^st^ (Least wealth) | 9.0 | (8.7-9.3) | 56.6 | (54.7-58.5) | 6.9 | (6.5-7.3) | 43.4 | (41.5-45.3) | 15.9 | (15.5-16.4) |
| 2^nd^ | 10.6 | (10.3-10.9) | 62.7 | (61.3-64.4) | 6.3 | (6.0-6.6) | 37.3 | (35.6-38.7) | 16.9 | (16.5-17.2) |
| 3^rd^ | 12.4 | (12.0-12.7) | 68.9 | (67.4-70.2) | 5.6 | (5.3-5.9) | 31.1 | (29.8-32.6) | 18.0 | (17.6-18.3) |
| 4^th^ | 14.3 | (13.8-14.7) | 74.5 | (72.5-76.1) | 4.9 | (4.5-5.3) | 25.5 | (23.9-27.5) | 19.2 | (18.6-19.7) |
| 5^th^ (Most wealth) | 16.3 | (15.5-16.9) | 79.5 | (77.4-81.2) | 4.3 | (3.8-4.7) | 21.0 | (18.8-22.6) | 20.5 | (19.7-21.2) |
| *Deprivation quintile* | | | | | | | | | | |
| 1^st^ (Most deprived) | 9.3 | (8.8-9.6) | 57.4 | (55.2-59.6) | 6.9 | (6.5-7.4) | 42.6 | (40.4-44.8) | 16.2 | (15.6-16.7) |
| 2^nd^ | 10.3 | (10.0-10.7) | 60.9 | (59.5-62.9) | 6.6 | (6.2-6.9) | 39.1 | (37.1-40.5) | 16.9 | (16.5-17.3) |
| 3^rd^ | 11.5 | (11.2-11.8) | 65.0 | (63.6-66.3) | 6.2 | (5.9-6.5) | 35.0 | (33.7-36.4) | 17.7 | (17.3-18.0) |
| 4^th^ | 12.7 | (12.4-13.1) | 68.6 | (67.2-70.2) | 5.8 | (5.4-6.1) | 31.4 | (29.8-32.8) | 18.5 | (18.1-18.9) |
| 5^th^ (Least deprived) | 14.0 | (13.5-14.6) | 72.2 | (70.6-74.2) | 5.4 | (5.0-5.8) | 27.8 | (25.8-29.4) | 19.4 | (18.8-20.0) |
| *Educational quintile* | | | | | | | | | | |
| Less than high school | 12.1 | (11.7-12.4) | 66.5 | (65.1-68.0) | 6.1 | (5.8-6.4) | 33.5 | (32.0-34.9) | 18.2 | (17.8-18.5) |
| High school | 13.7 | (13.1-14.2) | 70.6 | (68.5-72.6) | 5.7 | (5.3-6.1) | 29.4 | (27.4-31.5) | 19.4 | (18.7-20.0) |
| College or higher | 15.4 | (14.5-16.3) | 74.0 | (71.7-76.9) | 5.3 | (4.7-6.0) | 25.5 | (23.1-28.3) | 20.8 | (19.7-21.8) |
| *Marital status* | | | | | | | | | | |
| Not married | 11.1 | (10.8-11.5) | 63.4 | (62.1-65.2) | 6.3 | (6.0-6.7) | 36.0 | (34.8-37.9) | 17.5 | (17.1-17.9) |
| Married | 12.9 | (12.4-13.3) | 70.1 | (68.1-72.3) | 5.5 | (5.0-5.9) | 29.9 | (27.7-31.9) | 18.4 | (17.8-18.9) |

Life expectancies are stratified by socio-economic characteristics. Results are from a univariate analysis of each characteristic.

95% confidence intervals in parentheses.

| **S5 Table b: Age 70 frailty-free, frail and total life expectancies for men.** | | | | | | | | | | |
| --- | --- | --- | --- | --- | --- | --- | --- | --- | --- | --- |
|  | **Frailty-Free** | | | | **Frail** | | | | **Total** | |
| **Characteristic** | **Years** | | **Percentage** | | **Years** | | **Percentage** | | **Years** | |
| *All* | | | | | | | | | | |
|  | 11.7 | (11.4-12.1) | 76.0 | (74.9-77.6) | 3.7 | (3.4-3.9) | 24.0 | (22.4-25.1) | 15.4 | (15.0-15.7) |
| *Wealth quintile* | | | | | | | | | | |
| 1^st^ (Least wealth) | 9.0 | (8.6-9.4) | 66.2 | (63.4-68.5) | 4.6 | (4.2-5.0) | 33.8 | (31.5-36.6) | 13.6 | (13.1-14.0) |
| 2^nd^ | 10.3 | (10.0-10.6) | 71.5 | (70.0-73.3) | 4.1 | (3.8-4.3) | 28.5 | (26.7-30.0) | 14.4 | (14.0-14.7) |
| 3^rd^ | 11.7 | (11.4-12.0) | 77.0 | (75.4-78.1) | 3.5 | (3.3-3.8) | 23.0 | (21.9-24.6) | 15.2 | (14.9-15.5) |
| 4^th^ | 13.2 | (12.7-13.6) | 81.5 | (79.7-82.7) | 3.0 | (2.8-3.3) | 18.5 | (17.3-20.3) | 16.2 | (15.7-16.6) |
| 5^th^ (Most wealth) | 14.7 | (14.1-15.3) | 85.5 | (83.5-86.7) | 2.6 | (2.3-2.9) | 15.1 | (13.3-16.5) | 17.2 | (16.6-17.9) |
| *Deprivation quintile* | | | | | | | | | | |
| 1^st^ (Most deprived) | 9.3 | (8.8-9.7) | 67.9 | (65.4-70.1) | 4.4 | (4.0-4.8) | 32.1 | (29.9-34.6) | 13.7 | (13.2-14.2) |
| 2^nd^ | 10.3 | (10.0-10.6) | 71.5 | (69.9-73.4) | 4.1 | (3.8-4.4) | 28.5 | (26.6-30.1) | 14.4 | (14.0-14.8) |
| 3^rd^ | 11.4 | (11.1-11.7) | 75.5 | (73.9-76.6) | 3.8 | (3.5-4.0) | 25.2 | (23.4-26.1) | 15.1 | (14.8-15.5) |
| 4^th^ | 12.5 | (12.2-12.9) | 78.1 | (77.3-79.9) | 3.4 | (3.2-3.7) | 21.3 | (20.1-22.7) | 16.0 | (15.6-16.3) |
| 5^th^ (Least deprived) | 13.8 | (13.2-14.3) | 82.1 | (80.0-83.2) | 3.1 | (2.8-3.4) | 18.5 | (16.8-20.0) | 16.8 | (16.3-17.4) |
| *Educational quintile* | | | | | | | | | | |
| Less than high school | 11.8 | (11.5-12.1) | 76.1 | (74.8-77.5) | 3.7 | (3.5-3.9) | 23.9 | (22.5-25.2) | 15.5 | (15.2-15.8) |
| High school | 13.1 | (12.6-13.6) | 79.4 | (77.7-81.1) | 3.4 | (3.1-3.7) | 20.6 | (18.9-22.3) | 16.5 | (15.9-17.0) |
| College or higher | 14.4 | (13.6-15.2) | 82.3 | (79.8-84.5) | 3.1 | (2.7-3.6) | 17.7 | (15.5-20.2) | 17.5 | (16.6-18.4) |
| *Marital status* | | | | | | | | | | |
| Not married | 10.2 | (9.8-10.6) | 73.4 | (71.5-75.5) | 3.7 | (3.4-4.0) | 26.6 | (24.5-28.5) | 13.9 | (13.4-14.4) |
| Married | 12.4 | (12.0-12.8) | 77.0 | (75.7-78.7) | 3.7 | (3.4-3.9) | 23.0 | (21.3-24.3) | 16.1 | (15.7-16.5) |

Life expectancies are stratified by socio-economic characteristics. Results are from a univariate analysis of each characteristic.

95% confidence intervals in parentheses.

| S5 Table c: Age 70 frailty-free, frail, and total life expectancies in years for the most and least socioeconomically advantaged men and women. | | | | | | | | |
| --- | --- | --- | --- | --- | --- | --- | --- | --- |
|  | **Women (CI)** | | | | **Men (CI)** | | | |
|  | Years | | Percentage | | Years | | Percentage | |
| Most advantaged | | | | | | | | |
| **Frailty-free LE** | 17.2 | (16.4-17.9) | 81.1 | (80.4-82.1) | 15.6 | (14.9-16.3) | 86.7 | (86.6-87.8) |
| **Frail LE** | 4.1 | (3.6-4.5) | 19.3 | (17.9-19.6) | 2.3 | (2.1-2.7) | 12.8 | (12.2-13.4) |
| **Total LE** | 21.2 | (20.4-22.1) | - | - | 18.0 | (17.2-18.6) | - | - |
| Least advantaged | | | | | | | | |
| **Frailty-free LE** | 8.0 | (7.6-8.4) | 52.6 | (50.7-54.0) | 7.9 | (7.4-8.3) | 61.7 | (59.1-62.9) |
| **Frail LE** | 7.1 | (6.7-7.7) | 46.7 | (46.0-49.3) | 4.9 | (4.5-5.4) | 38.3 | (37.1-40.9) |
| **Total LE** | 15.2 | (14.6-15.7) | - | - | 12.8 | (12.2-13.3) | - | - |

The most advantaged group are people in both the most wealthy and least deprived quintiles. The least advantaged group are people in both the least wealthy and most deprived quintiles.

LE – life expectancy. CI – 95% confidence interval.

S6 Table: Hazard ratios and Bayesian Information Criterion (BIC) of models

| **S6 Table a:** Hazard ratios of the model with dependence on Age only. | | | |
| --- | --- | --- | --- |
|  |  | **Age (CI)** | |
| *Women* | | | |
| Robust–Prefrail |  | 1.04 | (1.04-1.04) |
| Robust–Death |  | 1.09 | (1.05-1.12) |
| Prefrail–Robust |  | 0.95 | (0.95-0.96) |
| Prefrail–Frail |  | 1.05 | (1.04-1.05) |
| Prefrail–Death |  | 1.11 | (1.09-1.13) |
| Frail–Prefrail |  | 0.98 | (0.98-0.99) |
| Frail–Death |  | 1.07 | (1.07-1.08) |
| *Men* | | | |
| Robust–Prefrail |  | 1.05 | (1.05-1.06) |
| Robust–Death |  | 1.08 | (1.05-1.11) |
| Prefrail–Robust |  | 0.97 | (0.96-0.97) |
| Prefrail–Frail |  | 1.05 | (1.04-1.05) |
| Prefrail–Death |  | 1.09 | (1.07-1.10) |
| Frail–Prefrail |  | 0.99 | (0.98-1.00) |
| Frail–Death |  | 1.07 | (1.06-1.07) |

Bayesian Information Criterion (BIC) women**:** 64 003; men: 52 083.

| **S6 Table b:** Hazard ratios of the model with covariates: Age, Wealth. | | | | |
| --- | --- | --- | --- | --- |
|  | **Age (CI)** | | **Wealth (CI)** | |
| *Women* | | | | |
| Robust–Prefrail | 1.04 | (1.04-1.04) | 0.88 | (0.86-0.90) |
| Robust–Death | 1.08 | (1.05-1.12) | 1.00 | (0.83-1.22) |
| Prefrail–Robust | 0.95 | (0.95-0.95) | 1.13 | (1.10-1.16) |
| Prefrail–Frail | 1.05 | (1.04-1.05) | 0.81 | (0.79-0.84) |
| Prefrail–Death | 1.11 | (1.10-1.13) | 0.86 | (0.78-0.96) |
| Frail–Prefrail | 0.98 | (0.98-0.99) | 1.17 | (1.12-1.23) |
| Frail–Death | 1.07 | (1.07-1.08) | 0.99 | (0.93-1.05) |
| *Men* | | | | |
| Robust–Prefrail | 1.06 | (1.05-1.06) | 0.88 | (0.85-0.90) |
| Robust–Death | 1.08 | (1.06-1.11) | 0.75 | (0.64-0.88) |
| Prefrail–Robust | 0.96 | (0.96-0.97) | 1.18 | (1.14-1.22) |
| Prefrail–Frail | 1.05 | (1.04-1.05) | 0.80 | (0.76-0.83) |
| Prefrail–Death | 1.09 | (1.08-1.10) | 0.99 | (0.91-1.08) |
| Frail–Prefrail | 0.99 | (0.98-1.00) | 1.08 | (1.01-1.15) |
| Frail–Death | 1.07 | (1.06-1.07) | 1.01 | (0.95-1.07) |

Wealth is categorised by quintile, with higher quintiles indicating greater wealth. Bayesian Information Criterion (BIC) women**:** 62 059; men: 50 735.

| **S6 Table c:** Hazard ratios of the model with covariates: Age, Educational attainment**.** | | | | |
| --- | --- | --- | --- | --- |
|  | **Age (CI)** | | **Education (CI)** | |
| *Women* | | | | |
| Robust–Prefrail | 1.04 | (1.03-1.04) | 0.89 | (0.86-0.93) |
| Robust–Death | 1.08 | (1.05-1.11) | 0.85 | (0.63-1.16) |
| Prefrail–Robust | 0.95 | (0.95-0.96) | 1.10 | (1.05-1.15) |
| Prefrail–Frail | 1.05 | (1.04-1.05) | 0.85 | (0.80-0.89) |
| Prefrail–Death | 1.11 | (1.09-1.13) | 0.89 | (0.77-1.04) |
| Frail–Prefrail | 0.98 | (0.98-0.99) | 1.11 | (1.04-1.18) |
| Frail–Death | 1.07 | (1.07-1.08) | 0.93 | (0.86-1.01) |
| *Men* | | | | |
| Robust–Prefrail | 1.05 | (1.05-1.06) | 0.90 | (0.86-0.94) |
| Robust–Death | 1.08 | (1.06-1.11) | 0.98 | (0.75-1.28) |
| Prefrail–Robust | 0.97 | (0.96-0.97) | 1.21 | (1.15-1.27) |
| Prefrail–Frail | 1.04 | (1.04-1.05) | 0.83 | (0.78-0.88) |
| Prefrail–Death | 1.09 | (1.07-1.10) | 0.96 | (0.85-1.08) |
| Frail–Prefrail | 0.99 | (0.98-1.00) | 1.00 | (0.92-1.09) |
| Frail–Death | 1.07 | (1.06-1.07) | 0.95 | (0.87-1.03) |

Education is categorised into three groups (<10 years, 10-11 years, and ≥12 years). Bayesian Information Criterion (BIC) women**:** 63 915; men: 52 000.

| **S6 Table d:** Hazard ratios of the model with covariates: Age, Area deprivation**.** | | | | |
| --- | --- | --- | --- | --- |
|  | **Age (CI)** | | **Deprivation (CI)** | |
| *Women* | | | | |
| Robust–Prefrail | 1.04 | (1.04-1.04) | 0.91 | (0.89-0.94) |
| Robust–Death | 1.08 | (1.05-1.12) | 1.01 | (0.78-1.29) |
| Prefrail–Robust | 0.95 | (0.95-0.96) | 1.11 | (1.08-1.15) |
| Prefrail–Frail | 1.05 | (1.04-1.05) | 0.88 | (0.85-0.90) |
| Prefrail–Death | 1.11 | (1.09-1.13) | 0.92 | (0.83-1.01) |
| Frail–Prefrail | 0.98 | (0.98-0.99) | 1.11 | (1.07-1.16) |
| Frail–Death | 1.07 | (1.07-1.08) | 0.97 | (0.93-1.02) |
| *Men* | | | | |
| Robust–Prefrail | 1.05 | (1.05-1.06) | 0.91 | (0.89-0.94) |
| Robust–Death | 1.08 | (1.06-1.11) | 0.73 | (0.62-0.86) |
| Prefrail–Robust | 0.96 | (0.96-0.97) | 1.16 | (1.12-1.20) |
| Prefrail–Frail | 1.05 | (1.04-1.05) | 0.85 | (0.82-0.89) |
| Prefrail–Death | 1.09 | (1.08-1.10) | 0.98 | (0.90-1.06) |
| Frail–Prefrail | 0.99 | (0.98-1.00) | 1.04 | (0.98-1.11) |
| Frail–Death | 1.07 | (1.06-1.07) | 1.00 | (0.94-1.05) |

Area deprivation is categorised by quintile, with higher quintiles indicating reduced area deprivation. Bayesian Information Criterion (BIC) women**:** 62 690; men: 51 752.

| **S6 Table e:** Hazard ratios of the model with covariates: Age, Marital status. | | | | |
| --- | --- | --- | --- | --- |
|  | **Age (CI)** | | **Married (CI)** | |
| *Women* | | | | |
| Robust-Prefrail | 1.04 | (1.03-1.04) | 0.85 | (0.79-0.91) |
| Robust-Dead | 1.08 | (1.04-1.11) | 0.68 | (0.39-1.19) |
| Prefrail-Robust | 0.95 | (0.95-0.96) | 1.06 | (0.98-1.16) |
| Prefrail-Frail | 1.04 | (1.04-1.05) | 0.80 | (0.73-0.87) |
| Prefrail-Dead | 1.11 | (1.09-1.12) | 0.83 | (0.62-1.10) |
| Frail-Prefrail | 0.98 | (0.98-0.99) | 1.07 | (0.94-1.21) |
| Frail-Dead | 1.08 | (1.07-1.08) | 1.10 | (0.94-1.29) |
| *Men* | | | | |
| Robust-Prefrail | 1.05 | (1.05-1.06) | 0.84 | (0.77-0.92) |
| Robust-Dead | 1.08 | (1.06-1.11) | 0.53 | (0.34-0.85) |
| Prefrail-Robust | 0.97 | (0.96-0.97) | 1.25 | (1.12-1.39) |
| Prefrail-Frail | 1.04 | (1.04-1.05) | 0.78 | (0.69-0.88) |
| Prefrail-Dead | 1.08 | (1.07-1.10) | 0.75 | (0.59-0.95) |
| Frail-Prefrail | 0.99 | (0.98-1.00) | 0.93 | (0.79-1.10) |
| Frail-Dead | 1.07 | (1.06-1.07) | 0.92 | (0.79-1.08) |

Marital status is categorised as married and not married, with not married as the reference category. Bayesian Information Criterion (BIC) women**:** 63 997; men: 52 051.

| **S6 Table f:** Hazard ratios of the model with covariates: Age, Wealth, Educational attainment. | | | | | | |
| --- | --- | --- | --- | --- | --- | --- |
|  | **Age (CI)** | | **Wealth (CI)** | | **Education (CI)** | |
| *Women* | | | | | | |
| Robust-Prefrail | 1.04 | (1.04-1.04) | 0.89 | (0.86-0.91) | 0.93 | (0.90-0.97) |
| Robust-Dead | 1.08 | (1.05-1.11) | 1.07 | (0.87-1.31) | 0.88 | (0.64-1.20) |
| Prefrail-Robust | 0.95 | (0.95-0.96) | 1.12 | (1.09-1.15) | 1.07 | (1.02-1.12) |
| Prefrail-Frail | 1.04 | (1.04-1.05) | 0.83 | (0.80-0.86) | 0.90 | (0.85-0.95) |
| Prefrail-Dead | 1.11 | (1.10-1.13) | 0.87 | (0.78-0.97) | 0.91 | (0.79-1.06) |
| Frail-Prefrail | 0.98 | (0.98-0.99) | 1.16 | (1.11-1.22) | 1.05 | (0.98-1.12) |
| Frail-Dead | 1.07 | (1.07-1.08) | 1.00 | (0.94-1.06) | 0.93 | (0.86-1.02) |
| *Men* | | | | | | |
| Robust-Prefrail | 1.05 | (1.05-1.06) | 0.88 | (0.86-0.91) | 0.94 | (0.90-0.98) |
| Robust-Dead | 1.09 | (1.06-1.11) | 0.75 | (0.63-0.89) | 1.08 | (0.82-1.43) |
| Prefrail-Robust | 0.96 | (0.96-0.97) | 1.16 | (1.12-1.20) | 1.14 | (1.08-1.20) |
| Prefrail-Frail | 1.05 | (1.04-1.05) | 0.81 | (0.78-0.85) | 0.90 | (0.84-0.96) |
| Prefrail-Dead | 1.09 | (1.07-1.10) | 0.99 | (0.91-1.09) | 0.96 | (0.85-1.09) |
| Frail-Prefrail | 0.99 | (0.98-1.00) | 1.08 | (1.01-1.16) | 0.97 | (0.89-1.06) |
| Frail-Dead | 1.07 | (1.06-1.07) | 1.02 | (0.96-1.09) | 0.93 | (0.85-1.02) |

Wealth is categorised by quintile, with higher quintiles indicating greater wealth. Education is categorised into three groups (<10 years, 10-11 years, and ≥12 years). Bayesian Information Criterion (BIC) women**:** 62 071; men: 50 751.

| **S6 Table g:** Hazard ratios of the model with covariates: Age, Wealth and Area deprivation. | | | | | | |
| --- | --- | --- | --- | --- | --- | --- |
|  | **Age (CI)** | | **Wealth (CI)** | | **Deprivation (CI)** | |
| *Women* | | | | | | |
| Robust-Prefrail | 1.04 | (1.04-1.04) | 0.89 | (0.87-0.91) | 0.94 | (0.92-0.97) |
| Robust-Dead | 1.08 | (1.04-1.12) | 1.24 | (0.94-1.64) | 0.92 | (0.70-1.21) |
| Prefrail-Robust | 0.95 | (0.95-0.95) | 1.10 | (1.07-1.14) | 1.07 | (1.04-1.11) |
| Prefrail-Frail | 1.05 | (1.04-1.05) | 0.84 | (0.81-0.87) | 0.93 | (0.89-0.96) |
| Prefrail-Dead | 1.11 | (1.10-1.13) | 0.85 | (0.76-0.95) | 0.96 | (0.87-1.06) |
| Frail-Prefrail | 0.98 | (0.98-0.99) | 1.15 | (1.09-1.20) | 1.07 | (1.03-1.13) |
| Frail-Dead | 1.08 | (1.07-1.08) | 1.00 | (0.94-1.07) | 0.97 | (0.92-1.02) |
| *Men* | | | | | | |
| Robust-Prefrail | 1.05 | (1.05-1.06) | 0.89 | (0.86-0.91) | 0.95 | (0.92-0.97) |
| Robust-Dead | 1.08 | (1.05-1.11) | 0.81 | (0.68-0.95) | 0.78 | (0.66-0.93) |
| Prefrail-Robust | 0.96 | (0.95-0.96) | 1.14 | (1.10-1.18) | 1.12 | (1.08-1.16) |
| Prefrail-Frail | 1.05 | (1.04-1.05) | 0.83 | (0.79-0.86) | 0.89 | (0.86-0.93) |
| Prefrail-Dead | 1.09 | (1.08-1.10) | 0.99 | (0.91-1.08) | 0.98 | (0.91-1.07) |
| Frail-Prefrail | 0.99 | (0.98-1.00) | 1.07 | (1.00-1.14) | 1.03 | (0.96-1.09) |
| Frail-Dead | 1.07 | (1.06-1.07) | 1.00 | (0.94-1.07) | 1.00 | (0.94-1.06) |

Wealth is categorised by quintile, with higher quintiles indicating greater wealth**.** Area deprivation is categorised by quintile, with higher quintiles indicating reduced area deprivation. Bayesian Information Criterion (BIC) women**:** 61 937; men: 50 565.

| **S6 Table h:** Hazard ratios of the model with covariates: Age, Wealth and Marital status**.** | | | | | | |
| --- | --- | --- | --- | --- | --- | --- |
|  | **Age (CI)** | | **Wealth (CI)** | | **Married (CI)** | |
| *Women* | | | | | | |
| Robust-Prefrail | 1.04 | (1.04-1.04) | 0.89 | (0.86-0.91) | 0.92 | (0.86-0.99) |
| Robust-Dead | 1.08 | (1.04-1.11) | 1.08 | (0.88-1.32) | 0.58 | (0.34-1.00) |
| Prefrail-Robust | 0.95 | (0.95-0.95) | 1.13 | (1.10-1.17) | 0.98 | (0.90-1.07) |
| Prefrail-Frail | 1.04 | (1.04-1.05) | 0.82 | (0.79-0.85) | 0.91 | (0.83-1.00) |
| Prefrail-Dead | 1.11 | (1.09-1.13) | 0.86 | (0.78-0.96) | 0.91 | (0.69-1.22) |
| Frail-Prefrail | 0.98 | (0.98-0.99) | 1.18 | (1.12-1.24) | 0.94 | (0.83-1.08) |
| Frail-Dead | 1.08 | (1.07-1.08) | 0.98 | (0.92-1.05) | 1.08 | (0.92-1.28) |
| *Men* | | | | | | |
| Robust-Prefrail | 1.05 | (1.05-1.06) | 0.88 | (0.86-0.90) | 0.87 | (0.79-0.94) |
| Robust-Dead | 1.08 | (1.05-1.11) | 0.77 | (0.65-0.91) | 0.56 | (0.35-0.90) |
| Prefrail-Robust | 0.96 | (0.96-0.97) | 1.17 | (1.13-1.21) | 1.14 | (1.02-1.27) |
| Prefrail-Frail | 1.05 | (1.04-1.05) | 0.80 | (0.77-0.84) | 0.85 | (0.75-0.96) |
| Prefrail-Dead | 1.09 | (1.07-1.10) | 1.00 | (0.92-1.09) | 0.74 | (0.59-0.94) |
| Frail-Prefrail | 0.99 | (0.98-1.00) | 1.09 | (1.02-1.16) | 0.87 | (0.73-1.03) |
| Frail-Dead | 1.07 | (1.06-1.07) | 1.01 | (0.95-1.08) | 0.93 | (0.80-1.09) |

Wealth is categorised by quintile, with higher quintiles indicating greater wealth**.** Marital status is categorised as married and not married, with not married as the reference category. Bayesian Information Criterion (BIC) women**:** 62 110; men: 50 742.

| **S6 Table i:** Hazard ratios of the model with covariates: Age, Wealth, Area deprivation and Educational attainment. | | | | | | | | |
| --- | --- | --- | --- | --- | --- | --- | --- | --- |
|  | **Age (CI)** | | **Wealth (CI)** | | **Deprivation (CI)** | | **Education (CI)** | |
| *Women* | | | | | | | | |
| Robust-Prefrail | 1.04 | (1.04-1.04) | 0.90 | (0.87-0.92) | 0.95 | (0.92-0.98) | 0.94 | (0.90-0.98) |
| Robust-Dead | 1.07 | (1.01-1.12) | 1.47 | (0.22-9.81) | 0.97 | (0.36-2.62) | 1.01 | (0.54-1.90) |
| Prefrail-Robust | 0.95 | (0.95-0.95) | 1.10 | (1.06-1.13) | 1.07 | (1.03-1.10) | 1.05 | (1.00-1.11) |
| Prefrail-Frail | 1.04 | (1.04-1.05) | 0.85 | (0.82-0.88) | 0.93 | (0.90-0.97) | 0.92 | (0.87-0.97) |
| Prefrail-Dead | 1.11 | (1.06-1.16) | 0.88 | (0.78-0.99) | 1.00 | (0.85-1.18) | 0.91 | (0.78-1.05) |
| Frail-Prefrail | 0.98 | (0.98-0.99) | 1.15 | (1.09-1.21) | 1.06 | (1.02-1.12) | 1.03 | (0.96-1.11) |
| Frail-Dead | 1.07 | (1.07-1.08) | 1.00 | (0.94-1.07) | 0.97 | (0.91-1.02) | 0.94 | (0.86-1.03) |
| *Men* | | | | | | | | |
| Robust-Prefrail | 1.05 | (1.05-1.06) | 0.89 | (0.87-0.92) | 0.95 | (0.92-0.98) | 0.95 | (0.91-0.99) |
| Robust-Dead | 1.09 | (1.06-1.11) | 0.79 | (0.66-0.93) | 0.78 | (0.65-0.92) | 1.14 | (0.86-1.49) |
| Prefrail-Robust | 0.96 | (0.96-0.97) | 1.12 | (1.08-1.16) | 1.11 | (1.07-1.15) | 1.12 | (1.06-1.18) |
| Prefrail-Frail | 1.05 | (1.04-1.05) | 0.84 | (0.80-0.88) | 0.90 | (0.86-0.94) | 0.92 | (0.86-0.98) |
| Prefrail-Dead | 1.09 | (1.08-1.10) | 1.00 | (0.91-1.09) | 0.99 | (0.91-1.07) | 0.98 | (0.86-1.11) |
| Frail-Prefrail | 0.99 | (0.98-1.00) | 1.07 | (1.00-1.15) | 1.03 | (0.97-1.10) | 0.96 | (0.88-1.06) |
| Frail-Dead | 1.07 | (1.06-1.07) | 1.01 | (0.95-1.08) | 1.00 | (0.95-1.06) | 0.93 | (0.85-1.01) |

Wealth is categorised by quintile, with higher quintiles indicating greater wealth**.** Area deprivation on categorised by quintile, with higher quintiles indicating reduced area deprivation. Educational attainment is categorised into three groups (<10 years, 10-11 years, and ≥12 years). Bayesian Information Criterion (BIC) women**:** 61 979; men: 50 600.

| **S6 Table j:** Hazard ratios of the model with covariates: Age, Wealth, Area deprivation, and Marital status**.** | | | | | | | | |
| --- | --- | --- | --- | --- | --- | --- | --- | --- |
|  | **Age (CI)** | | **Wealth (CI)** | | **Deprivation (CI)** | | **Married (CI)** | |
| *Women* | | | | | | | | |
| Robust-Prefrail | 1.04 | (1.03-1.04) | 0.90 | (0.87-0.92) | 0.95 | (0.92-0.98) | 0.93 | (0.86-1.01) |
| Robust-Dead | 1.06 | (0.98-1.16) | 1.45 | (0.11-18.82) | 0.96 | (0.25-3.70) | 0.75 | (0.20-2.83) |
| Prefrail-Robust | 0.95 | (0.94-0.95) | 1.10 | (1.07-1.14) | 1.07 | (1.04-1.11) | 0.97 | (0.89-1.06) |
| Prefrail-Frail | 1.04 | (1.04-1.05) | 0.84 | (0.81-0.88) | 0.92 | (0.89-0.96) | 0.92 | (0.83-1.01) |
| Prefrail-Dead | 1.11 | (1.04-1.19) | 0.89 | (0.78-1.01) | 1.02 | (0.79-1.33) | 0.98 | (0.60-1.59) |
| Frail-Prefrail | 0.98 | (0.98-0.99) | 1.16 | (1.10-1.22) | 1.07 | (1.03-1.12) | 0.95 | (0.83-1.08) |
| Frail-Dead | 1.07 | (1.06-1.09) | 0.98 | (0.91-1.05) | 0.95 | (0.90-1.02) | 1.06 | (0.88-1.26) |
| *Men* | | | | | | | | |
| Robust-Prefrail | 1.05 | (1.05-1.06) | 0.89 | (0.86-0.91) | 0.95 | (0.92-0.98) | 0.88 | (0.81-0.96) |
| Robust-Dead | 1.08 | (1.05-1.11) | 0.81 | (0.68-0.95) | 0.80 | (0.67-0.96) | 0.62 | (0.39-0.99) |
| Prefrail-Robust | 0.96 | (0.95-0.96) | 1.13 | (1.09-1.17) | 1.12 | (1.07-1.16) | 1.11 | (0.99-1.24) |
| Prefrail-Frail | 1.05 | (1.04-1.05) | 0.83 | (0.79-0.87) | 0.90 | (0.86-0.94) | 0.88 | (0.78-1.00) |
| Prefrail-Dead | 1.09 | (1.07-1.10) | 1.00 | (0.92-1.09) | 1.00 | (0.92-1.09) | 0.74 | (0.58-0.93) |
| Frail-Prefrail | 0.99 | (0.98-1.00) | 1.08 | (1.01-1.15) | 1.04 | (0.97-1.10) | 0.86 | (0.73-1.03) |
| Frail-Dead | 1.07 | (1.06-1.07) | 1.01 | (0.94-1.07) | 1.00 | (0.94-1.06) | 0.95 | (0.81-1.12) |

Wealth is categorised by quintile, with higher quintiles indicating greater wealth**.** Area deprivation is categorised by quintile, with higher quintiles indicating reduced area deprivation. Marital status is categorised as married and not married, with not married as the reference category. Bayesian Information Criterion (BIC) women**:** 62 004; men: 50 587.

| **S6 Table k:** Hazard ratios of the model with covariates: Age, Wealth, Area deprivation, Educational attainment, and Marital status**.** | | | | | | | | | | |
| --- | --- | --- | --- | --- | --- | --- | --- | --- | --- | --- |
|  | **Age (CI)** | | **Wealth (CI)** | | **Deprivation (CI)** | | **Education (CI)** | | **Married (CI)** | |
| *Women* | | | | | | | | | | |
| Robust-Prefrail | 1.04 | (1.03-1.04) | 0.90 | (0.88-0.93) | 0.95 | (0.93-0.98) | 0.94 | (0.90-0.98) | 0.93 | (0.86-1.00) |
| Robust-Dead | 1.07 | (1.04-1.11) | 1.15 | (0.92-1.43) | 0.85 | (0.68-1.06) | 0.89 | (0.65-1.23) | 0.62 | (0.35-1.07) |
| Prefrail-Robust | 0.95 | (0.95-0.95) | 1.10 | (1.07-1.13) | 1.07 | (1.03-1.10) | 1.05 | (1.00-1.10) | 0.97 | (0.89-1.06) |
| Prefrail-Frail | 1.04 | (1.04-1.05) | 0.85 | (0.82-0.89) | 0.94 | (0.90-0.97) | 0.91 | (0.87-0.96) | 0.92 | (0.83-1.01) |
| Prefrail-Dead | 1.11 | (1.09-1.13) | 0.88 | (0.78-0.98) | 0.99 | (0.89-1.10) | 0.91 | (0.78-1.06) | 0.91 | (0.68-1.21) |
| Frail-Prefrail | 0.98 | (0.98-0.99) | 1.15 | (1.09-1.21) | 1.07 | (1.02-1.12) | 1.03 | (0.96-1.10) | 0.95 | (0.83-1.08) |
| Frail-Dead | 1.08 | (1.07-1.08) | 1.00 | (0.94-1.07) | 0.97 | (0.92-1.03) | 0.94 | (0.87-1.03) | 1.08 | (0.91-1.27) |
| *Men* | | | | | | | | | | |
| Robust-Prefrail | 1.05 | (1.05-1.06) | 0.89 | (0.87-0.92) | 0.95 | (0.93-0.98) | 0.95 | (0.91-0.99) | 0.88 | (0.81-0.97) |
| Robust-Dead | 1.09 | (1.06-1.11) | 0.79 | (0.66-0.93) | 0.80 | (0.67-0.95) | 1.14 | (0.86-1.50) | 0.62 | (0.39-0.99) |
| Prefrail-Robust | 0.96 | (0.96-0.97) | 1.12 | (1.08-1.16) | 1.11 | (1.06-1.15) | 1.11 | (1.05-1.18) | 1.10 | (0.98-1.23) |
| Prefrail-Frail | 1.05 | (1.04-1.05) | 0.84 | (0.80-0.88) | 0.91 | (0.87-0.95) | 0.92 | (0.86-0.98) | 0.89 | (0.78-1.01) |
| Prefrail-Dead | 1.09 | (1.07-1.10) | 1.00 | (0.92-1.10) | 1.00 | (0.92-1.09) | 0.98 | (0.87-1.12) | 0.73 | (0.58-0.93) |
| Frail-Prefrail | 0.99 | (0.98-1.00) | 1.08 | (1.01-1.16) | 1.04 | (0.97-1.11) | 0.96 | (0.88-1.06) | 0.86 | (0.73-1.03) |
| Frail-Dead | 1.07 | (1.06-1.07) | 1.02 | (0.95-1.09) | 1.01 | (0.95-1.07) | 0.93 | (0.85-1.01) | 0.96 | (0.82-1.12) |

Wealth is categorised by quintile, with higher quintiles indicating greater wealth**.** Area deprivation is categorised by quintile, with higher quintiles indicating reduced area deprivation. Education is categorised into three groups (<10 years, 10-11 years, and ≥12 years). Marital status is categorised as married and not married, with not married as the reference category. Bayesian Information Criterion (BIC) women**:** 62 020; men: 50 624.

S7 Figure: Comparison of total life expectancy estimates to Office for National Statistics estimates

| **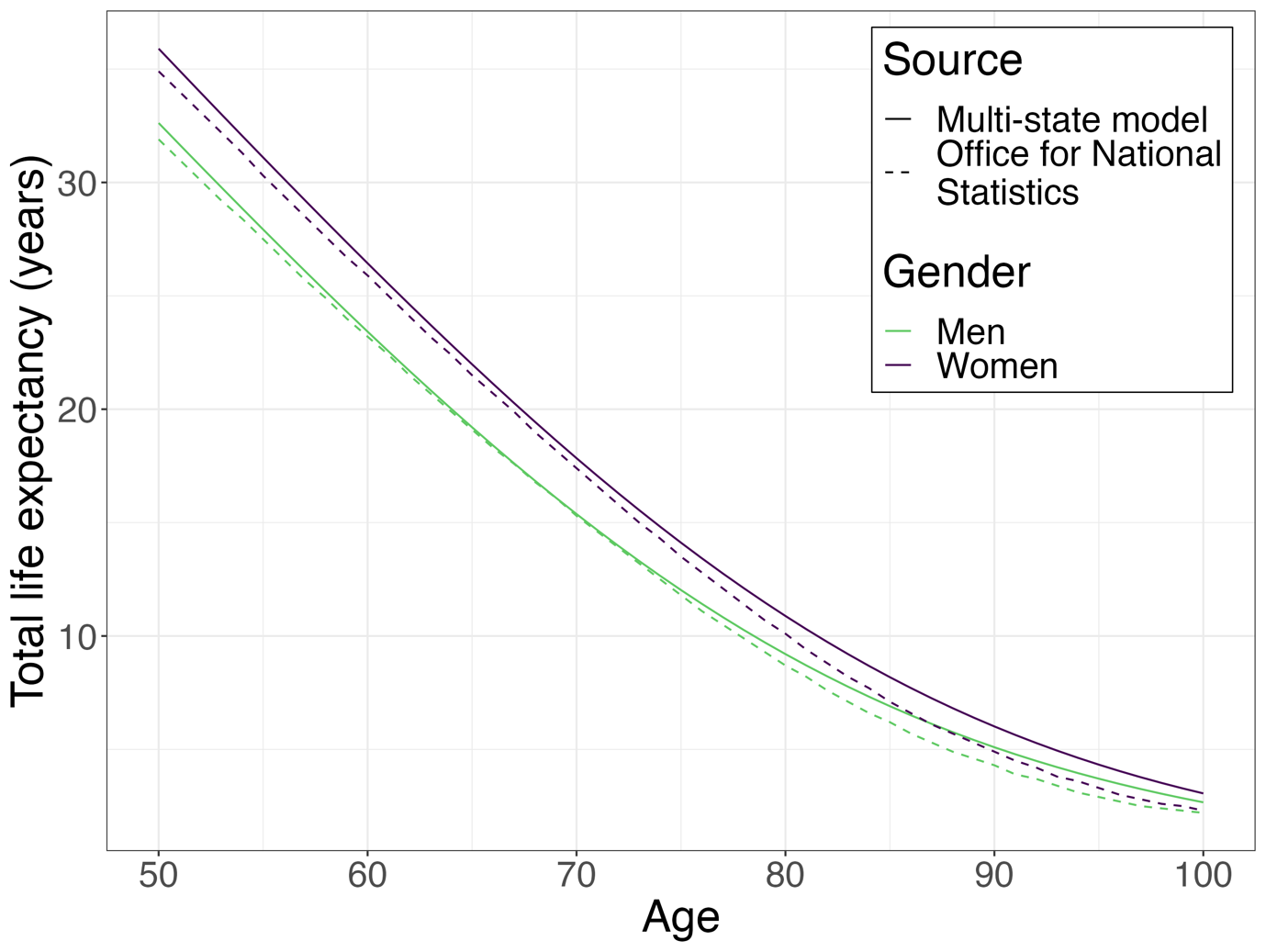** |
| --- |
| Comparison of total life expectancy estimates in years from the multi-state model used in this study and the Office for National Statistics life tables for England, 2019 [42]. |
